# Interdisciplinary Management of Traumatic Injuries to the Kidneys and Urinary Tract Caused by Blunt Abdominopelvic Trauma

**DOI:** 10.3390/jcm13195765

**Published:** 2024-09-27

**Authors:** Johann J. Wendler, Christian Albert, Hannes Cash, Frank Meyer, Maciej Pech, Martin Schostak, Peter R. Mertens, Markus Porsch

**Affiliations:** 1 University Clinic for Urology, Urooncology, Robot-Assisted and Focal Therapy, University Hospital Magdeburg A.ö.R., Medical Faculty of the Otto-von-Guericke-University Magdeburg, Leipziger Str. 44, 39120 Magdeburg, Germany; hannes.cash@med.ovgu.de (H.C.); martin.schostak@med.ovgu.de (M.S.); markus.porsch@med.ovgu.de (M.P.); 2 Urology Practice, 39104 Magdeburg, Germany; 3 University Clinic for Cardiology and Angiology, University Hospital Magdeburg A.ö.R., Medical Faculty of the Otto-von-Guericke University Magdeburg, Leipziger Straße 44, 39120 Magdeburg, Germany; 4 Department of Nephrology, Central Clinic Bad Berka, Robert-Koch-Allee 9, 99438 Bad Berka, Germany; 5 Urology Practice, 10117 Berlin, Germany; 6 University Clinic for General, Visceral, Vascular and Transplantation Surgery, University Hospital A.ö.R., Medical Faculty of Otto-von-Guericke-University Magdeburg, Leipziger Straße 44, 39120 Magdeburg, Germany; frank.meyer@med.ovgu.de; 7 University Clinic for Radiology and Nuclear Medicine, University Hospital A.ö.R., Medical Faculty of Otto-von-Guericke-University Magdeburg, Leipziger Straße 44, 39120 Magdeburg, Germany; maciej.pech@med.ovgu.de; 8 University Clinic for Nephrology and Hypertension, Diabetes and Endocrinology, University Hospital A.ö.R., Medical Faculty of Otto-von-Guericke-University Magdeburg, Leipziger Straße 44, 39120 Magdeburg, Germany; peter.mertens@med.ovgu.de

**Keywords:** polytrauma, kidney trauma, urological injury, acute kidney injury, risk assessment, contrast-enhanced imaging-associated AKI

## Abstract

**Purpose:** Blunt abdominopelvic trauma frequently results in injuries to the urinary organs, especially in polytrauma. The urotrauma is rarely an acute life-threatening event; however, it may lead to severe complications. **Methods:** This review addresses the under-representation of urological trauma management in interdisciplinary medical training and its impact on patient outcomes. It compiles evidence-based recommendations and guidelines from multiple specialties, focusing on common challenges in managing these injuries. The resource is tailored for primary care physicians in radiology, trauma surgery, internal medicine, urology, and nephrology. **Results:** Urinary tract injuries can occur even if the patient’s condition initially appears normal. An exclusion diagnosis is obligatory by contrast medium tomography of the entire urinary tract and, if suspected, an additional uroendoscopic examination. Interventional therapy by catheterisation of the urinary tract is often required. Urosurgical treatment is not commonly needed, but when there is a demand, it must be administered via an interdisciplinary approach with visceral and trauma surgery. Over 90% of life-threatening kidney injuries (usually up to grade 4–5 AAST) are presently treated by interventional radiologists. Acute kidney injury (AKI) as a complication in trauma patients may complicate clinical management and often worsens the outcome. The incidence of trauma-associated AKI in patients admitted to an intensive care unit is high. **Conclusions:** Patients suffering from blunt abdominopelvic trauma should ideally be referred to certified trauma centres with subspecialised or fully specialised care provided by visceral/vascular surgery, trauma surgery, interventional radiology, urology, and nephrology. This recommendation is based on the complex nature of most damage patterns.

## 1. Introduction

Blunt abdominopelvic trauma and polytrauma present complex diagnostic and management challenges, especially when life-threatening conditions like haemorrhages, haematomas, and shock create a blurred clinical picture. While primary care often focuses on stabilising vital functions and controlling bleeding, urological injuries can easily be overlooked if not explicitly considered, leading to incorrect initial treatments and worse patient outcomes. Although urotraumas, apart from severe renal bleeding, are rarely immediately life threatening, they can cause severe complications and chronic functional limitations if not properly managed. The early involvement of urologists is essential for planning appropriate interdisciplinary treatment. Additionally, nephrologists should be involved in cases where there is a risk of acute kidney dysfunction. Blunt abdominal trauma is common in road traffic accidents and falls, often involving the urinary system. Complications like intraperitoneal ruptures can lead to urinary peritonitis and disrupt the pelvic integrity, while bone fragments may cause tissue damage and provoke phlegmon. Early examination and specialised care are crucial to improving survival rates and reducing complications.

## 2. Purpose of This Review

This narrative review offers an in-depth overview of the standard interdisciplinary management of primary injuries to the kidneys, ureters, urinary bladder, and, in men, the pelvic and posterior urethra, particularly in the context of blunt abdominopelvic trauma. Despite the importance of managing urological trauma, this area is often under-represented in interdisciplinary medical training, which can impact patient outcomes. To address this gap, this review compiles evidence-based recommendations and guidelines on the standard care of these injuries, drawing from various medical specialties. It focuses on the most common and significant challenges associated with such trauma. This resource is specifically designed for primary care physicians in radiology, trauma surgery, general and internal medicine, urology, and nephrology. It serves as a quick reference and an educational tool for interdisciplinary teaching, particularly aimed at the training of resident physicians.

## 3. Background

### Epidemiology

According to the 2022 annual report of the TraumaRegister DGU^®^ (TR-DGU) of the Deutsche Gesellschaft für Unfallchirurgie (German Society for Trauma Surgery), out of the 35,747 seriously injured people recorded, 14% experienced polytrauma and 54% were at least seriously injured, of which 69% were men. Blunt trauma accounted for the majority (96%), although blunt abdominal trauma only rarely occurs in isolation. Together, the involvement of the abdomen including the flank and pelvis was around 30%, i.e., with a potential injury to the urinary organs. In total, 89% were treated regionally or nationally, i.e., at centres with access to interdisciplinary urological care [1]. About 10% of abdominopelvic traumas involve injury to the urological organs. Kidney trauma occurs in around 5% of all trauma cases [2]. Ureter injuries are rare (1%); of these, one third are caused by blunt trauma, usually from traffic accidents with extreme deceleration as well as hyperextension and hyperlordosis, and usually affect the upper ureter [3]. Bladder trauma occurs in pelvic contusions or blunt-object force trauma in the lower abdomen, with blunt bladder injuries being associated with pelvic fractures in 60–90% of cases, and with other intra-abdominal injuries in 44–68% of cases [4]. A bladder injury is associated with a urethral injury in 5–20% of cases [5]. Overall, kidney injuries represent the most common urological trauma (50% overall, children ≥90%; 20% mono- and 80% polytrauma; [6,7]).

## 4. Guidelines

The following selected guidelines are currently available as the primary guidelines for injuries to the urinary organs:

- European guideline, EAU Guidelines on Urological Trauma from the European Association of Urology (Ed. March 2023; [8]);
- German S3 guidelines for the treatment of polytrauma or severe injury (German Society for Accident Surgery (AWMF [Association of Scientific Medical Societies], version 4.0, 31 December 2022; [9]), as well as the S2k guidelines for polytrauma care in children (AWMF, version 2.1, 10/2020 [7]);
- US Urotrauma Guideline of the American Urological Association (AUA) [10], the World Society of Emergency Surgery (WSES), and the American Association for the Surgery of Trauma (AAST) Kidney and Urogenital Trauma Management Guidelines [11,12].

The following selected sources are currently available as the primary guidelines for the assessment of kidney function and damage and the use of contrast media in patients with kidney disease:

- Kidney Disease: Improving Global Outcomes (KDIGO) Acute Kidney Injury Work Group. KDIGO clinical practice guideline for acute kidney injury, 2012 [13];
- Recommendations on Acute Kidney Injury Biomarkers from the Acute Disease Quality Initiative (ADQI) Consensus Conference: A Consensus Statement, 2020 [14];
- Use of Intravenous Iodinated Contrast Media in Patients with Kidney Disease: Consensus Statements from the American College of Radiology and the National Kidney Foundation, 2020 [15].

## 5. Classifications

The most commonly used descriptive classification is the organ-specific AAST Organ Injury Scoring Scale (Organ Injury Scale; AAST-OIS) of the American Association for the Surgery of Trauma (Table 1, Table 2, Table 3 and Table 4; [11]). In addition, the World Society of Emergency Surgery (WSES) classifies kidney injuries with additional consideration of the patient’s haemodynamic, bleeding-related status (Table 5; [12]). The severity of a polytrauma (general and Berlin polytrauma definition) using the Injury Severity Score (ISS) and the Abbreviated Injury Scale (AIS) classification does not permit conclusions on the pattern of urological injury. Until proven otherwise, an uncertain course of events must therefore be assumed to have resulted in severe abdominal trauma and polytrauma, and thus also in injury to urological organs. Regarding the type of violence, a distinction can be made between blunt abdominal trauma (shocks, falls, contusions, crushing, overstretching, whiplash, and deceleration trauma) and explosive abdominal trauma (pressure or barotrauma caused by explosions or implosions), whereby combinations of these, including penetration (open) trauma, are also possible. Surgical treatment of non-urological, complex injury patterns also carries the risk of iatrogenic trauma to the urinary organs [8].

## 6. General Management

### 6.1. Initial Evaluation

Blunt abdominopelvic trauma is potentially life threatening, but urological injuries on their own are rarely acutely life threatening. Frankly, this only applies to severe kidney injuries of AAST-OIS grade 4–5 (Table 1) or WSES grade 4–5 (Table 5 [8,11,12]). To identify such a severe trauma, the anamnesis of the course of events, the targeted recording of the symptoms, and an in-depth primary diagnosis all play a pivotal part in deciding the relative priorities of specialist medical measures. Significant symptoms of injuries to the urinary organs are bruises, haematomas, and abrasions; swelling of the abdomen, pelvis, and perineal area, flank and back as well as abdominal, pelvic, back, and flank pain; defensive tension or acute abdomen; gross haematuria or bleeding from the urethra; urinary urgency and urinary retention; anal or vaginal bleeding; digitorectal status (dislocation of the prostate, haematoma); an atypical digitovaginal status (space-occupying swelling); and obvious signs of a pelvic, rib, or spine fracture and even circulation instability. However, a normal status does not exclude a relevant urological injury [8,10].

### 6.2. Damage Control

In addition to the initial necessity to stabilise circulation, the control of bleeding and intensive-medical first aid, some temporary urological interventions are applicable for urinary diversion (ureteral splinting/upper urinary diversion, suprapubic, and/or transurethral bladder catheter). Catheterisation, as well as extraluminal drainage and abdominopelvic packing, is used in primary trauma surgery. A time-consuming, reconstructive urological operation can then be carried out following stabilisation of the patient or at the beginning of convalescence [8,9,10].

### 6.3. Urinary Bladder Catheterisation

Transurethral urinary bladder catheterisation is often outlined in the literature as part of the immediate emergency ward management in the emergency room. However, this does not provide any clinical advantage in immediate primary care in cases with potential lower urotraumas. Rather, it carries the risk of additional, iatrogenic traumatisation in the event of an existing and potentially unrecognised urethral or bladder injury such as urethral perforation, rupture, dislocation, extraluminal placement, or false passage, which may complicate urological reconstruction. In the event of a pelvic ring fracture, injury to the urethra and bladder must be excluded as they may occur in up to 70% of cases. The absence of bleeding from the urethra or an inconspicuous perineal or anal area do not constitute a clinical exclusion criterion [16]. In such cases, non-expert bladder catheterisation should initially be avoided. Gross haematuria or bleeding from the urethra after catheterisation, when the clinical and anatomical condition is still unclear, always raises the question of causality and traumatic catheter insertion [17]. However, it is safest to wait for primary imaging to rule out a lower urinary tract injury.

If there is no suspicion of a urethral injury, a transurethral catheter insertion can be attempted by experienced urological specialists. A simple passage and prompt urine drainage without gross haematuria render additional iatrogenic damage unlikely [8]. Alternatively, retrograde urethrography or a urethrocystogram, possibly with catheterisation, can be carried out in the emergency room or in emergency traumatological surgical treatment. If an emergency visceral surgical laparotomy must be carried out, a suprapubic catheter should—if possible—be inserted for diagnostic and therapeutic purposes or, if longer-term provision is needed, to reduce the risk of a urethral stricture [8,10].

### 6.4. Imaging

As part of the emergency-room treatment, according to the concept of “advanced trauma life support” (ATLS), a standardised set of quasi-simultaneous measures is carried out to stabilise and secure vital parameters and functions, as is a diagnostic “primary survey” immediately after physical and sonographic examinations, according to the “enhanced-focused-assessment-with-sonography-for-trauma” (eFAST) protocol [10]. However, the eFAST-Sono and the urological emergency sonography should be regarded as exploratory examinations as part of the prioritisation. On account of their strong examiner dependence and poor sensitivity, every 5th–10th organ lesion is not detected sonographically. Therefore, a sonography is not considered sufficient in primary diagnosis, and computed tomography (CT) is recommended for all patients [10,18].

An emergency-room CT is the standard examination in the initial emergency-room phase (performed on average within 25 min of admission); an in-depth uroradiological diagnostic should be carried out [8,10]. To rule out urotrauma, a multi-slice spiral CT should be performed, including but not limited to the entire abdomen and pelvis including the perineal area and external genitals, to sequence the entire urinary tract. For the urological examination, an initial series before the administration of contrast-enhancement medium (CM) is recommended, to allow for the potential differentiation of radiopaque foreign bodies in the urinary tract that may include bone fragments, urolithiasis, or accidental and iatrogenic foreign bodies from possible CM accumulations or recesses [6,8,19]. Subsequently, after the CM administration of 100–150 mL of iodine-containing CM 350 mg/mL, 3–5 mL/s, administered through a peripheral vein, a series of images in the arterial CM phase is recorded using bolus tracking 25–40 s after CM administration, and in the portal venous CM phase 70–80 s after CM administration, respectively [19]; this is obligatory for a urological assessment of the urinary outflow tract in the late venous urogram phase, performed 7–20 min after CM administration. In the latter, the entire upper urinary tract, including the renal pelvic calyx system and the ureters up to the immediately prevesical area with the beginning of bladder opacification, should be visualised as a passage to rule out CM or urine leakage.

With the bladder catheter in place, a CT cystogram with 300–350 mL of diluted CM should be performed to rule out or to classify a bladder injury. A passive low filling in the urogram phase with the catheter clamped is insufficient [8,10]. In unstable patients, a late CM phase or urography may initially have to be avoided; this can then be carried out intraoperatively under fluoroscopy, or by performing another CT shortly after stabilisation.

Intraoperative ultrasound (IOUS) is a diagnostic imaging technique used during surgical procedures to provide the real-time visualisation of internal structures, which enhances the surgeon’s ability to perform precise and targeted interventions (real-time decision making). Currently, intraoperative ultrasound (IOUS) is only used to a limited extent in emergency trauma surgery and has not yet established a firm role. In urological emergency procedures of interdisciplinary trauma surgery, it is primarily applied in percutaneous urinary drainage (e.g., drainage, nephrostomy, and cystostomy) and for assessing testicular status.

### 6.5. Free Fluid/Urine Extralumination

As part of the differential diagnostic/therapeutic assessment, the determination of the creatinine concentration from aspirates/drainages of free fluid retention is a cornerstone measurement in cases of suspected urinoma. It is important to use the uniform parameter unit of creatinine in the serum and from aspirates/drainages (recommended SI unit: μmol/L). Urine has a creatinine concentration approximately 80 times higher compared to serum, so that elevated creatinine values in the aspirate will imply urine extralumination and thus injury to the urinary organs [6].

Larger intraperitoneal and retroperitoneal accumulations of extraluminally leaked urine (uroperitoneum) can, in addition to the clinical symptoms of an acute abdomen and paralytic ileus, lead to the (re)absorption of urinary and renally excreted substances. This can cause the paraclinical condition of renal failure, including an increase in serum creatinine and metabolic acidosis.

### 6.6. Recommendation on the Early Diagnostic and Management of Acute Kidney Injury

Acute renal dysfunction as a complication in trauma patients may complicate management and outcome. The recent literature indicates that the incidence of trauma-associated acute kidney injury (AKI) in patients admitted to an intensive care unit (ICU) is as high as 20–24%. Approximately 10% of those patients who develop AKI will require the induction of kidney replacement therapy (KRT), corresponding to approximately 2% of the total trauma population [20,21].

In general, causes of post-traumatic AKI are multifactorial. Circulatory shock associated with major trauma is most frequently due to haemorrhage, and it may lead to inadequate renal perfusion [22]. The most common clinical presentation in patients with suggested shock includes tachycardia, hypotension, obtundation, or unconsciousness. Patients may present with pallor or mottled skin as a sign of anaemia or circulatory centralisation, and with tachypnoea as a reaction to pain or as a mechanism of compensation to metabolic acidosis and hyperlactatemia. Anuria or oliguria and hypothermia may be present already in the early phases of shock. The traditional classification of severity is performed using the Glasgow Coma Scale.

An early kidney status assessment in these patients at risk may therefore draw attention onto potential modifiable factors that contribute to AKI development and progression. In the initial 24 h, acute kidney dysfunction may present as asymptomatic or subclinical. It may involve a combination of prerenal and intrarenal dysfunction or pathology, in addition to less common postrenal urinary obstruction. In cases of reduced urine output (oliguria to anuria), extralumination and urinary obstruction must always be ruled out. Dark-coloured concentrated urine (provided there is no macrohematuria) suggests a possible volume deficit, or indicates crush syndrome (myoglobulinuria due to severe traumatic rhabdomyolysis). Of critical importance is the circulatory situation and the associated renal perfusion to prevent (hypovolemic) shock (due to bleeding or volume deficit), SIRS (Systemic Inflammatory Response Syndrome), sepsis (due to secondary infections), and cardiopulmonary decompensation.

While traditional nephrological involvement has focused on intensive care and the convalescence phase following initial treatment, the implementation of novel laboratory diagnostic options, such as biomarkers and consented interventional care bundles, has shifted nephrologists’ attention to the early care phase in the emergency department, as discussed in the following paragraphs.

#### 6.6.1. Laboratory Values

For the diagnostic assessment and classification of kidney function, consensus definitions are based on the elevation of serum creatinine (SCr) and urinary output (UO) [13], both of which are of limited sensitivity and specificity, specifically in cases with trauma-associated shock or potential urinary flow obstructions due to ruptures or bleeding. Therefore, the early detection of acute renal functional decline and detection of tissue stress or injury before the onset of SCr/UO-based AKI may be of particular importance.

Cystatin C is an alternative established marker of kidney function that is not depending on muscle mass and dietary intake, which therefore offers an alternative approach to estimate GFR [23]. Formulas using serum cystatin C alone and in combination with serum creatinine are available and may further improve the precision of estimated GFR [24]. Additionally, an assessment of emerging biomarkers for the detection of potential subclinical organ-associated tissue damage such as in missed clinical injury or the early onset of haemorrhagic shock [25] may lead to additional diagnostics and adjustment of care.

Elevated urinary or plasma biomarker levels associated to cell stress, iron metabolism, or inflammation were recently found to be of additional value to rule in or rule out patients at an elevated risk for adverse renal outcomes after cardiac or major abdominal surgery [26,27]. AKI associated with conditions with reduced renal perfusion, such as shock or during the process of ischemia-reperfusion injury, was frequently associated with elevation in levels of neutrophil gelatinase-associated lipocalin (NGAL). Considering several known confounders regarding the diagnostic accuracy of NGAL for the prediction of AKI, involving 26 studies with >6650 patients including 23% AKI events, recently clinical applicable NGAL cutoff values to rule in or out an elevated risk of AKI were derived [28]. Urinary and plasma NGAL levels were determined, and the predictive capability for severe AKI was compared with markers of cell cycle arrest such as TIMP-2 and IGFBP7 (tissue inhibitor of metalloproteinases-2 and insulin-like growth factor-binding protein 7) [29]. In a recent study including polytrauma patients, [TIMP-2]·[IGFBP7] was found to be of aid to identify patients with tubular damage that subsequently evolves into a clinically apparent AKI [30].

The Kidney Disease Improving Global Outcome (KDIGO)-classification criteria of AKI are currently undergoing revision and are suggested and expected to include biomarker-based definitions of AKI phenotypes that are not characterised by elevated SCr or a decline in urine output (UO), but they acknowledge an early rise of kidney biomarkers above the agreed thresholds (Table 6) [14]. Patients with biomarker positivity, in the absence of an SCr increase, are attributed to have subclinical AKI [31]. The recognition of these AKI phenotypes was driven by the understanding that such patients are at an elevated risk for adverse kidney-related events, such as the need for renal replacement therapy initiation and an elevated risk of mortality. In patients admitted to the emergency department or intensive care unit, biomarker positivity therefore adds prognostic information and may contribute to a refined clinical risk assessment [32,33,34] and the subsequent adjustment of therapy. It is suggested that the combination of damage and functional biomarkers, along with clinical information, be used to improve the diagnostic accuracy of AKI, to recognise the different pathophysiological processes, to discriminate AKI aetiology, and to assess AKI severity [14].

Negative biomarker test results may point toward ruling out imminent risk. Sequential measurements, however, may be relevant according to the course of patients’ risk profiles [14,29]. In Table 6, acknowledging the use of biomarkers, we present the staging criteria of AKI as recommended by the Acute Disease Quality Initiative (ADQI) [14].

Traditional and established assessments of point-of-care urine dipstick status, urine sediment evaluation, and the quantification of proteinuria are cost-effective, but follow-up assessments are advisable to exclude false-negative or false-positive findings in cases of urinary tract involvement in trauma with intraluminal bleeding, oligo-anuria or extravasation regarding their sensitivity and specificity for genuine acute, chronic, and nephritic kidney diseases.

#### 6.6.2. Kidney Care Bundles

The implementation of early goal-directed management protocols based on biomarkers may improve the awareness of AKI risk, and may consecutively positively affect or mitigate the course of AKI [35,36]. The suggested optimisation of care [13] is based on the amelioration of circulatory distress, optimisation of volume status, discontinuation of nephrotoxic medication, and cautious use of contrast agents, which we address in a separate paragraph. In a multicentre randomised controlled trial, the adherence to the KDIGO bundle recommendations was associated with a significant reduction in the incidence of moderate to severe AKI in high-risk patients undergoing cardiac surgery [37].

Specifically, after major abdominal surgery, an early biomarker-based assessment of AKI and the implementation of KDIGO-care bundles accordingly reduced AKI severity, postoperative SCr increase, and the length of stay in the intensive care unit and in hospital [27]. An early consultation with a nephrologist in patients with AKI is recommended, and it may positively influence the course of AKI regarding the frequency of complications and thus improve patients’ outcomes [38]. Despite these encouraging results, recent observational data, however, indicate that in routine clinical practice, compliance to the recommended patient management in accordance with the KDIGO recommendations was low [39], addressing the need for the improvement of standard clinical care procedures.

In summary, early biomarker-guided AKI diagnostic and implementation of care bundles are readily available and demonstrated superiority regarding kidney-associated outcomes in multiple settings [40]. Due to the acute trauma, the cause of AKI may not be completely preventable. Future prospective studies focussing on the early detection and prevention of worsening or sustained AKI in this specific population are warranted.

#### 6.6.3. Use of Radiographic Contrast Agents in Critically Ill Patients with Risk of AKI

The risk assessment and interpretation of the association of contrast agent-induced AKI have recently been subject to critical reflection, and a revised guideline is published in a joint statement by the American College of Radiology and the National Kidney Foundation to improve and standardise care [15].

In brief, the risk for AKI due to contrasting radiographic agents was found to be overrated, specifically due to the confounding of conventional risk factors of AKI and the study design with a lack of control groups leading to the mixing of contrast-associated acute kidney injury with contrast-induced AKI in uncontrolled studies [15].

Kidney function screening is indicated to identify patients at a high risk for AKI. A personal history of kidney disease (chronic kidney disease, remote AKI, kidney surgery, or ablation) is the strongest risk factor, but also age, diabetes mellitus, hypertension, and proteinuria are regarded as potential risk factors indicating the need for a kidney function assessment. Prophylaxis is indicated for patients who have AKI or an eGFR less than 30 mL/min/1.73 sqm and are not undergoing maintenance dialysis. Volume expansion is the preferred method, and when administered, the individual risks of heart failure and other hypervolemic conditions should be critically considered [15].

Adequate trauma diagnosis and care should be given priority, and contrast agents should not be withheld based on kidney function. However, reducing the amount of contrast agent may be considered in all patients, if possible, while maintaining the optimal diagnostic procedure is feasible [15]. Despite popular belief, kidney replacement therapy (KRT) should not be initiated, or maintenance dialysis plans adjusted solely based on contrast media administration [15,41,42].

In clinical application, there seem to be no relevant differences in contrast induced-AKI risk between iso-osmolality and low-osmolality iodinated contrast media [43]; however, the cessation of nonessential nephrotoxic medication is recommended in patients at risk when feasible [15].

### 6.7. Documentation

Of 90,962 patients with an Injury Severity Score ≥ 16 (TraumaRegister DGU^®^, between 2009 and 2016), 5.9% (*n* = 5345) had suffered GU injuries [44]. Since trauma to the urinary organs often leads to litigation (e.g., for accident insurance, occupational accident insurance, health insurance, social insurance, legal protection insurance, liability insurance, pension insurance, etc.), it is important for legal reasons to ensure unambiguous documentation [1,17,44,45]. Owing to the interdisciplinary nature of cases, separate departmental urological documentation should always be performed, in addition to the central documentation. In late complications such as chronic renal impairment, its secondary ailments are assessed individually and in total: hypertension, anaemia, polyneuropathy, and osteopathy; damage to the urinary tract (urinary stasis, recurrent urinary tract infections, chronic inflammation, micturition disorders, and catheterisations); and incontinence, fistula formation, and artificial urinary diversion. Their causality is also assessed and documented as either causally related damage or subsequent, non-related damage [17].

## 7. Special Management

### 7.1. Injuries to the Kidney, the Renal Pelvic Calyx System, and the Vessels Supplying the Kidneys

Kidney injuries due to blunt abdominal trauma occur as a consequence of associated deceleration and/or blunt flank trauma [6,46].

Non-surgical management, consisting of conservative measures and radiological intervention using digital subtraction angiography (DSA) and selective angioembolisation (SAE), is now considered the therapy of choice, because of its higher kidney preservation rate and lower invasiveness [8,10,47,48,49,50]. The success rate of SAE is up to 95% for AAST-3, up to 90% for AAST-4, and up to 76% for AAST-5 [10,50,51]. In the event of secondary bleeding, repeated SAE achieves a kidney preservation rate of 67% [51]. If traumatic dissection and occlusion of the distal renal artery segment occur, vascular stenting may be considered; however, if this procedure leads to severe bleeding without the option of SAE, then proximal embolisation of the main trunk is necessary [52].

Renal pelvic calyx system ruptures with urinoma usually require urgent diversion using ureter splinting, nephrostomy if necessary, and perirenal drainage [6,8,10,46].

At the time of writing, there is only one urological surgical indication for surgical treatment in cases of circulatory instability that cannot be stabilised by mass transfusion and intensive care measures (WSES grade 4) with severe vascular injuries to the main renal vessels and active bleeding (AAST grade 5) without immediate interventional radiology (DSA, SAE), as well as in cases of immediate emergency laparotomy indication due to multiple injuries to other organs in polytrauma [8,10]. The median transperitoneal approach to the renal pedicle vessels is chosen before opening the renal fascia in order to avoid decompression and additional mass bleeding of the kidney. In such cases, primary nephrectomy is usually attempted [10].

The average (necessary) nephrectomy rate during surgical exploration is very high, at 30% [53]. Singular kidney ruptures that need to be reconstructed can be adapted (renography); pure kidney pole fragments can be treated, if necessary, by partial kidney/pole resection, incision, and patch plasty [6,8,10,46]. Stable perirenal haematomas detected during surgical exploration because of other organ injuries should not be opened [8].

### 7.2. Injuries to the Ureter

Ureteral injuries are often initially overlooked, mainly owing to an incomplete diagnosis (80% of diagnoses are delayed) [46]. Unexceptional findings from the CT urogram—or, if necessary, excretory urography—should be checked by retrograde ureteropyelography with the option of immediate transurethral retrograde ureter splinting. Alternatively, one can use percutaneous renal puncture, nephrostomy, and antegrade pyeloureterography, or consider the option of immediate or second-stage antegrade ureter splinting [8,10]. In addition, trauma-related haematomas and oedema in the abdomen and retroperitoneum may lead to an abdominal compartment syndrome, which in turn can lead inter alia to the compression of the kidneys and ureters with consequent reduced blood flow, possible urinary stasis, and intrarenal plus postrenal kidney failure. In addition to surgical pressure relief by laparotomy and haematoma removal, ureter splinting is recommended for drainage and protection.

Partial ureteral lesions (AAST grade 1–3) should be treated as quickly as possible after stabilisation using the above-mentioned ureteral splinting techniques, to reduce the acute risk of complications from urinoma, urinary stasis, and sepsis, along with the long-term risk of strictures. If necessary, this must be completed with ureterorenoscopic support. In the event of an emergency surgical exploration of the retroperitoneum, open ureter splinting and the primary suture of the leak site may be considered [8,54,55]. In the case of complete ruptures and long-distance defects (AAST grade 4–5), a specific reconstruction is necessary, depending on the location and grade of the lesion: ureteroureterostomy (≤3 cm), ureteropyeolo- or calicostomy (proximal, close to the renal pelvis), transureteroureterostomy (upper and middle third), ureter reimplantation, if necessary with psoas hitch (≤6 cm, lower third) or if necessary with Boari plastic (≤15 cm distance, middle third), ileum interposition (up to the entire ureter) and, if necessary, iliac/pelvic kidney autotransplantation (complete middle and lower third) [6,8,46]. A primary ureteroureterostomy with an end-to-end anastomosis or ureteral reimplantation should be attempted in stable patients as part of an exploratory laparotomy because of the more favourable prognosis (tissue conditions) [8,10]. Extensive lesions and reconstructions usually require a delayed procedure after convalescence and with temporary nephrostomy diversion. A ureter ligation may be necessary until reconstruction or as part of the nephrostomy treatment. The Detour system is available as a heterologous, elective ureter replacement [56].

### 7.3. Injuries to the Urinary Bladder

Intraperitoneal bladder ruptures occur due to blunt abdominal trauma with a full bladder and the rupture of the peritoneally covered part of the bladder. They require the fastest possible operative exposure, urinoma drainage, and suturing of the defect to avoid secondary complications such as peritonitis, sepsis, or paralytic ileus [5,57]. Extraperitoneal bladder ruptures (70%, 5% combined) usually occur related to pelvic fractures, and they are often due to the impalement of the anterolateral bladder wall with bone fragments, causing bleeding or gross haematuria [4]. Most extraperitoneal ruptures can be treated conservatively by urinary catheter drainage [8,10,37,58]. Protruding bone fragments require the reduction or removal of the bone fragments and, if necessary, adaptation of the bladder wall, usually as part of initial emergency surgical treatment (osteosynthesis, fixator) [4,6]. Follow-up monitoring is carried out by cystogram via the existing permanent bladder catheter.

### 7.4. Injuries to the Posterior Pelvic Urethra

In the area of the membranous urethra, the urethra is firmly attached to the urogenital diaphragm of the pelvic floor. Blunt injuries to the posterior urethra arise almost exclusively from pelvic fractures resulting from blunt abdominal pelvic trauma, most often located in particular on the anterior pelvic ring, usually due to traffic accidents. Owing to the shear forces acting on the urethra and the pelvic floor, in men, rupturing usually occurs above the membranous urethra (supradiaphragmal) at the transition from the prostatic urethra to the pars membranacea urethrae (pelvic floor, external urethra sphincter), and more rarely below i.e., infradiaphragmatic. A distinction is made between partial and complete ruptures (complete longitudinal and lateral dislocation of the urethral ends; no longer a continuous lumen).

Injuries to the prostate or the prostatic urethra are extremely rare and usually occur in the anterior midline (commissure). Posterior (proximal) urethral injuries are not immediately life threatening, but they lead to high morbidity because of strictures and often to incontinence and erectile dysfunction due to the concomitant injury of the periprostatic/periurethral vascular nerve bundles.

Partial posterior urethral injuries in men should preferably be splinted with a transurethral catheter until healing. If this is not possible and continuity is maintained, a temporary suprapubic urinary bladder catheter diversion (cystostomy) is sufficient. In cases of complete avulsion, primary retrograde splinting is attempted (urographically/fluoroscopically guided and, if necessary, endoscopic), and if necessary additionally antegrade suprapubic transvesical using the Rendezvous procedure (transurethral threading of the urethral stump with the prostatic urethra and urinary bladder, “immediate” or “early re-alignment”) in order to significantly reduce the reconstruction rate and risk for stricture. If this is not possible, a cystostomy (suprapubic urinary catheter) is performed in the interests of damage control. Primary reconstruction in the acute phase (within 48 h) is not recommended, as this attempt inherits a greater risk of bleeding, erectile dysfunction, urinary stress incontinence, and strictures [8,46]. Early reconstruction (after 2 days to 6 weeks) offers no advantage over delayed reconstruction (“delayed urethroplasty” after 3–6 months; [8,46]).

Urethral injuries in women are extremely rare because of the short urethra, the absence of a prostate, and the normally high flexibility/elasticity of the vagina, urethra, and pelvic floor [8]. Here, a distinction is made between longitudinal or partial injuries and transverse or complete injuries, although vaginal involvement may be present, which carries the risk of a later urethrovaginal fistula. In most cases, primary reconstruction is not possible, so the cystostomy is performed before early reconstruction [8,46].

## 8. Discussion

Trauma management has evolved significantly over the past decade. While emergency surgeries were once often unavoidable for severe injuries, particularly kidney trauma, there is now a growing reliance on interventional radiology and conservative approaches. Angioembolisations for haemorrhage control and non-operative management of high-grade renal injuries have become well-established practices. The future holds great potential in the application of artificial intelligence (AI) in diagnostics, allowing for earlier and more precise injury detection, and in creating more individualised and efficient treatment algorithms. AI is increasingly being integrated into diagnostic imaging, particularly in the interpretation of CT scans for trauma patients. AI algorithms, including deep learning models, can assist radiologists by enhancing the detection of subtle injuries that might be missed during manual assessment, such as minor renal lacerations or microhaemorrhages. These technologies improve diagnostic accuracy, reduce interpretation time, and support clinical decision making by predicting the likelihood of complications based on imaging features. The use of AI in CT diagnostics is particularly promising for rapid triage and management decisions in emergency settings, allowing for early and precise interventions [59,60]. Hybrid operating rooms combine surgical and imaging capabilities, allowing for immediate diagnostic and therapeutic interventions in a single setting. This setup is particularly advantageous in managing complex urological traumas and enabling simultaneous endovascular procedures (such as angioembolisation) and open or laparoscopic surgery. Hybrid ORs improve patient outcomes by reducing the time between diagnosis and treatment, facilitating real-time adjustments during surgery, and minimising the need for patient transfers between the radiology suite and operating room. The use of hybrid ORs represents a shift towards more integrated and efficient trauma care, supporting advanced techniques like combined embolisation and the surgical repair of kidney injuries [61]. There is an ongoing debate regarding the optimal management of high-grade renal trauma, particularly in haemodynamically stable patients. While guidelines generally favour non-operative management, recent studies have raised questions about the role of routine follow-up imaging and the timing of interventions like angioembolisation. Some argue that early intervention with angioembolisation can reduce complications and the need for nephrectomy, while others advocate for a more conservative approach with observation and delayed intervention if necessary [62]. Recent research highlights the growing role of artificial intelligence (AI) and machine learning in predicting and managing acute kidney injury (AKI). These technologies are being used to identify high-risk patients early, guide personalised treatment strategies, and optimised dosing during AKI management [63,64].

## 9. Limitations

**Figure A1:**
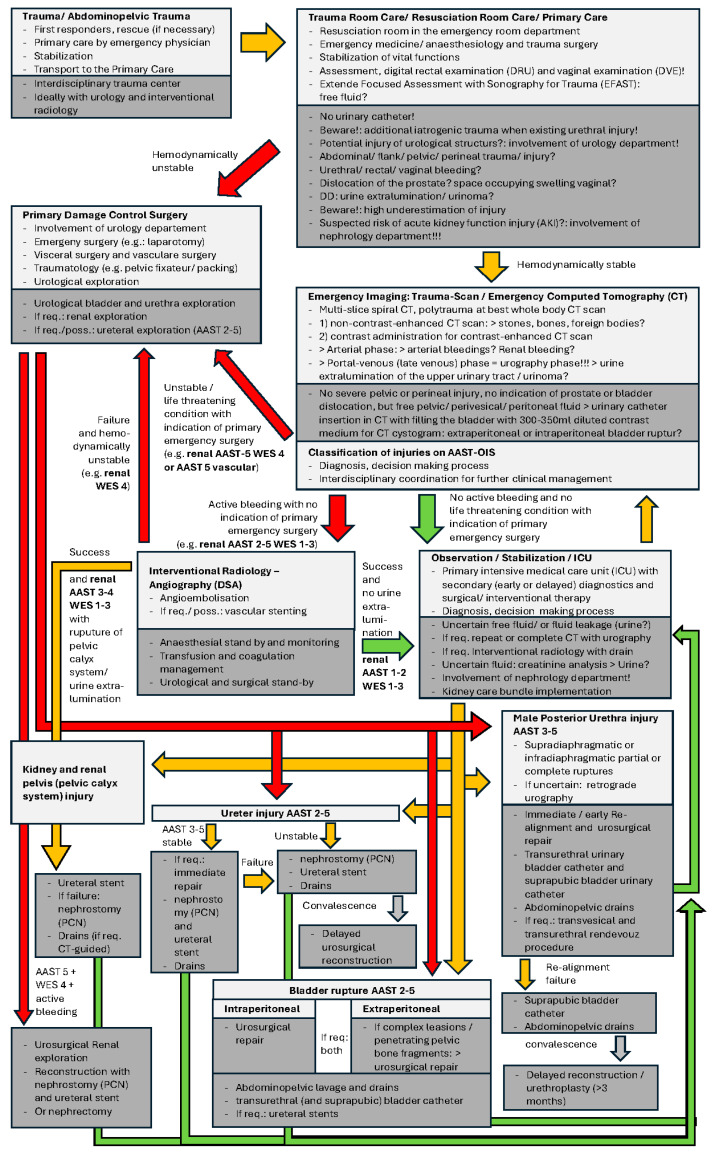
Flowchart schema for interdisciplinary management of traumatic injuries to the kidneys and urinary tract caused by blunt abdominopelvic trauma.

The limitations of this publication concern several aspects of methodology and data access: the work is a narrative review without a systematic meta-analysis. The focus is on selected major German and English-language guidelines and narrative summaries, which results in a lack of deeper, discipline-specific discussion and detailed analysis of the guidelines. This limitation may lead to a skewed representation of current practice. Insurance and registry data are unfortunately not freely accessible, restricting the analysis of the actual incidence and treatment outcomes. This makes it difficult to provide accurate statements about the frequency and long-term success of treatments in clinical practice. This publication exclusively focuses on blunt trauma involving urological organs, primarily highlighting the urological perspective in interdisciplinary management. This limits the transferability of the results to other types of trauma, such as penetrating injuries or those involving other organs. Although the interdisciplinary aspect is emphasised, the focus is mainly on urological interventions, which impairs a comprehensive representation of interdisciplinary management, especially by non-urological specialties. The practical aspects presented in the text are challenging to analyse; therefore, a flowchart schema is employed to facilitate quicker understanding. Most available flowcharts are tailored individually for specific organs or situations, often containing limited information or emphasising specialised delayed reconstruction techniques. However, in clinical practice, emergency patients frequently present with potentially concurrent injury patterns. Consequently, we have developed a detailed, comprehensive flowchart schema. Please refer to Appendix A Figure A1 for further details.

## 10. Practical Conclusions

- Urinary tract injuries due to abdominal trauma are common, but they are often primarily asymptomatic. In all cases, an explicit exclusion diagnosis using native and multiphase contrast-medium computed tomography with a late urogram phase of the entire abdomen/pelvis is obligatory.
- The most commonly used descriptive classification is the organ-specific AAST Organ Injury Scoring Scale (Organ Injury Scale; AAST-OIS) of the American Association for the Surgery of Trauma.
- As part of the differential diagnostic/therapeutic assessment, the determination of the creatinine concentration from aspirates/drainages of free fluid retention is a cornerstone measurement in cases with suspected urinoma.
- Posterior urethral injuries are primarily associated with pelvic fractures and are often associated with high morbidity and incontinence. Catheterisation without excluding a urethral lesion entails a risk of additional trauma. Therefore, emergency catheter insertion without prior retrograde urethrography should be avoided. If primary urological interventional transurethral katheterisation is not possible, cystostomy and a delayed reconstruction must be performed.
- A cystogram should be performed to rule out bladder injury. Intraperitoneal bladder ruptures must be surgically fixed with high urgency; extraperitoneal bladder ruptures may often be cured by using a bladder catheter.
- Ureter splinting as early as possible is advantageous in cases of lesions. For extensive defects elective, specific reconstruction is usually necessary.
- In cases of kidney injury, non-operative management by interventional radiological angioembolisation is the first-line therapy due to its higher organ and kidney function preservation rate. Over 90% of life-threatening kidney injuries (usually up to grade 4–5 AAST) are, nowadays, treated by interventional radiologists. The nephrectomy rate during surgical exploration is very high, reaching 30%.
- An assessment of kidney function (GFR, proteinuria) is recommended in trauma patients at risk of AKI. In patients at risk, an early assessment for acute kidney injury using clinical information in addition to kidney function improves kidney-associated outcomes. Traditional measures of kidney function include SCr, SCr-based estimated glomerular filtration rate (eGFR) equations, and urine output. An assessment of Cystatin C eGFR may improve the assessment of renal function. Formulas to estimate GFR including both SCr and Cystatin C are available.
- The prophylaxis of contrast-associated AKI by volume expansion is indicated in patients at high risk. Individual patient comorbidities should be critically considered. Kidney replacement therapy (KRT) should not be initiated to remove contrast medium.

## Figures and Tables

**Table 1:** AAST-OIS kidney classification.

AAST-OIS Grade ^a^	Type of Injury	Pattern of Injury
1 ^a^	Haematoma and/or contusion	Subcapsular, constant-size haematoma, and/or parenchymal contusion without parenchymal rupture, micro- or macrohaematuria, urological status normal
2 ^a^	Haematoma	Perirenal haematoma of constant size limited to the renal fascia
	Laceration	Cortical parenchymal rupture ≤1.0 cm in depth without urine extralumination
3	Laceration	Parenchymal rupture >1.0 cm in depth without rupture of the renal pelvic calyx system and without urine extralumination
		Any parenchymal vascular injury (interlobar artery/arcuatae renis) or any active bleeding within the renal fascia
4	Laceration	Parenchymal tear extending into the renal pelvic calyx system with urine extralumination; a rupture of the renal pelvis and/or complete avulsion of the pyeloureteral junction
	Vascular	Segmental artery or segmental vein injury
		Active bleeding via renal fascia into the retroperitoneum or peritoneum
		Segmental or complete renal infarction due to vascular thrombosis without active bleeding
5	Laceration	Shattered kidney with loss of recognisable parenchymatous renal anatomy
	Vascular	Rupture of the main renal artery or vein
		Avulsion of the renal hilum; devascularised kidney with active bleeding

^a^ For bilateral injuries up to grade 3, upgrade by one level.

**Table 2:** AAST-OIS ureter classification.

AAST-OIS Grade ^a^	Type of Injury	Pattern of Injury
1 ^a^	Haematoma	Contusion or haematoma without devascularisation and without urine extralumination
2 ^a^	Laceration	Transection <50% of the circumference, urine extralumination
3	Laceration	Transection ≥50% of the circumference, urine extralumination
4	Laceration	Complete transection with <2 cm of devascularisation
5	Laceration	Complete avulsion with >2 cm of devascularisation, discontinuity/dislocation

^a^ For bilateral injuries up to grade 3, upgrade by one level.

**Table 3:** AAST-OIS bladder classification.

AAST-OIS Grade ^a^	Type of Injury	Pattern of Injury ^b^
1 ^a^	Haematoma	Contusion, intramural haematoma (of the bladder wall)
	Laceration	Partial tear in bladder wall thickness, not continuous
2 ^a^	Laceration	Extraperitoneal bladder wall rupture <2 cm, extraperitoneal urine extralumination
3	Laceration	Extraperitoneal bladder wall rupture ≥2 cm, extraperitoneal urine extralumination
		Intraperitoneal bladder wall rupture <2 cm, intraperitoneal urine extralumination
4	Laceration	Intraperitoneal bladder wall rupture ≥2 cm, intraperitoneal urine extralumination
5	Laceration	Intraperitoneal or extraperitoneal bladder wall rupture extending into the bladder neck or the trigone/ureteral ostium

^a^ For bilateral injuries up to grade 3, upgrade by one level. ^b^ Combined urinary bladder injury from extraperitoneal and intraperitoneal rupture possible.

**Table 4:** AAST-OIS urethra classification in men.

AAST-OIS Grade ^a^	Type of Injury	Pattern of Injury (Determined by Retrograde Urethrocystography)
1 ^a^	Contusion	Bleeding from the meatus urethrae, without extraluminate
2 ^a^	Stretch injury	Elongation of the urethra, without extraluminate
3	Partial rupture	Extraluminate from the urethral lesion, but with contrast transfer into the urinary bladder
4	Complete rupture	Urethral dislocation <2 cm, extraluminate without transfer of CM into the urinary bladder
5	Complete rupture	Urethral dislocation ≥2 cm or extension into the prostate, extraluminate without CM transfer into the urinary bladder

CM, contrast medium. ^a^ For bilateral injuries up to grade 3, upgrade by one level.

**Table 5:** WSES kidney injury classification.

WSES Grade	AAST-OIS Grade ^a^	Haemodynamic Situation
1 ^a^	1–2	Stable
2 ^a^	3 or segmental vascular injury	
3	4–5 or any type of parenchymal lesion with dissection or occlusion of major vessels	
4	Any (1–5)	Unstable (short-term stabilisation may be possible)

WSES, World Society of Emergency Surgery. ^a^ For bilateral injuries up to AAST grade 3, upgrade by one level.

**Table 6:** Staging criteria recommended by the Acute Disease Quality Initiative (ADQI), 2020.

Functional Criteria	Stage	Damage Criteria
No change or SCr level increase <0.3 mg/dL, and no UO criteria	1S	Biomarker positive
Increase in SCr level by ≥0.3 mg/dL for ≤48 h or ≥150% for 7 days and/or UO <5 mL/kg/h for >6 h	1A	Biomarker negative
	1B	Biomarker positive
Increase in SCr level by >200% and/or UO <5 mL/kg/h for >12 h	2A	Biomarker negative
	2B	Biomarker positive
Increase in SCr level by >300% (≥4.0 mg/dL with an acute increase of ≥0.5 mg/dL) and/or UO <0.3 mL/kg/h for >24 h or anuria for >12 hand/or acute KRT	3A	Biomarker negative
	3B	Biomarker positive

Readily available markers of kidney function include serum creatinine (SCr), Cystatin C, and urine output (UO). KRT, kidney replacement therapy.
